# Supplementary material for: Unveiling the Bioactive Potential of the Invasive Jellyfish Phyllorhiza punctata Through Integrative Transcriptomic and Proteomic Analyses
Source: Biomolecules. 2025 Aug 4;15(8):1121. doi: 10.3390/biom15081121 (PMC12383608; doi:10.3390/biom15081121)
Supplement: Supplementary file 1 [file biomolecules-15-01121-s001.zip › Suplementary Material/FileS3/PEPFOLD4/XP_065066977_5065/clusters.html]

 PEP-FOLD clusters


Download archive of all models  here   

|  |  |  |
| --- | --- | --- |
| # | file | sOPEP |
| # cluster 1 - Size: 4 |  |  |
|  | XP\_065066977\_5065-model1 | -16.4389 |
|  | XP\_065066977\_5065-model1.1 | -16.4389 |
|  | XP\_065066977\_5065-model1.2 | -16.4389 |
|  | XP\_065066977\_5065-model1.3 | -16.4389 |
| # cluster 2 - Size: 85 |  |  |
|  | XP\_065066977\_5065-model2 | -16.1055 |
|  | XP\_065066977\_5065-model2.1 | -16.1055 |
|  | XP\_065066977\_5065-model2.2 | -16.1055 |
|  | XP\_065066977\_5065-model2.3 | -16.1055 |
|  | XP\_065066977\_5065-model2.4 | -16.1055 |
|  | XP\_065066977\_5065-model2.5 | -16.1055 |
|  | XP\_065066977\_5065-model2.6 | -16.1055 |
|  | XP\_065066977\_5065-model2.7 | -16.1055 |
|  | XP\_065066977\_5065-model2.8 | -16.1055 |
|  | XP\_065066977\_5065-model2.9 | -16.1055 |
|  | XP\_065066977\_5065-model2.10 | -16.1055 |
|  | XP\_065066977\_5065-model2.11 | -16.1055 |
|  | XP\_065066977\_5065-model2.12 | -16.1055 |
|  | XP\_065066977\_5065-model2.13 | -16.1055 |
|  | XP\_065066977\_5065-model2.14 | -16.1055 |
|  | XP\_065066977\_5065-model2.15 | -16.1055 |
|  | XP\_065066977\_5065-model2.16 | -16.1055 |
|  | XP\_065066977\_5065-model2.17 | -16.1055 |
|  | XP\_065066977\_5065-model2.18 | -16.1055 |
|  | XP\_065066977\_5065-model2.19 | -16.1055 |
|  | XP\_065066977\_5065-model2.20 | -16.1055 |
|  | XP\_065066977\_5065-model2.21 | -16.1055 |
|  | XP\_065066977\_5065-model2.22 | -16.1055 |
|  | XP\_065066977\_5065-model2.23 | -16.1055 |
|  | XP\_065066977\_5065-model2.24 | -16.1055 |
|  | XP\_065066977\_5065-model2.25 | -16.1055 |
|  | XP\_065066977\_5065-model2.26 | -16.1055 |
|  | XP\_065066977\_5065-model2.27 | -16.1055 |
|  | XP\_065066977\_5065-model2.28 | -16.1055 |
|  | XP\_065066977\_5065-model2.29 | -16.1055 |
|  | XP\_065066977\_5065-model2.30 | -16.1055 |
|  | XP\_065066977\_5065-model2.31 | -16.1055 |
|  | XP\_065066977\_5065-model2.32 | -16.1055 |
|  | XP\_065066977\_5065-model2.33 | -16.1055 |
|  | XP\_065066977\_5065-model2.34 | -16.1055 |
|  | XP\_065066977\_5065-model2.35 | -16.1055 |
|  | XP\_065066977\_5065-model2.36 | -16.1055 |
|  | XP\_065066977\_5065-model2.37 | -16.1055 |
|  | XP\_065066977\_5065-model2.38 | -16.1055 |
|  | XP\_065066977\_5065-model2.39 | -16.1055 |
|  | XP\_065066977\_5065-model2.40 | -16.1055 |
|  | XP\_065066977\_5065-model2.41 | -16.1055 |
|  | XP\_065066977\_5065-model2.42 | -16.1055 |
|  | XP\_065066977\_5065-model2.43 | -16.1055 |
|  | XP\_065066977\_5065-model2.44 | -16.1055 |
|  | XP\_065066977\_5065-model2.45 | -16.1055 |
|  | XP\_065066977\_5065-model2.46 | -16.1055 |
|  | XP\_065066977\_5065-model2.47 | -16.1055 |
|  | XP\_065066977\_5065-model2.48 | -16.1055 |
|  | XP\_065066977\_5065-model2.49 | -16.1055 |
|  | XP\_065066977\_5065-model2.50 | -16.1055 |
|  | XP\_065066977\_5065-model2.51 | -16.1055 |
|  | XP\_065066977\_5065-model2.52 | -16.1055 |
|  | XP\_065066977\_5065-model2.53 | -16.1055 |
|  | XP\_065066977\_5065-model2.54 | -16.1055 |
|  | XP\_065066977\_5065-model2.55 | -16.1055 |
|  | XP\_065066977\_5065-model2.56 | -16.1055 |
|  | XP\_065066977\_5065-model2.57 | -16.1055 |
|  | XP\_065066977\_5065-model2.58 | -16.1055 |
|  | XP\_065066977\_5065-model2.59 | -16.1055 |
|  | XP\_065066977\_5065-model2.60 | -16.1055 |
|  | XP\_065066977\_5065-model2.61 | -16.1055 |
|  | XP\_065066977\_5065-model2.62 | -16.1055 |
|  | XP\_065066977\_5065-model2.63 | -16.1055 |
|  | XP\_065066977\_5065-model2.64 | -16.1055 |
|  | XP\_065066977\_5065-model2.65 | -16.1055 |
|  | XP\_065066977\_5065-model2.66 | -16.1055 |
|  | XP\_065066977\_5065-model2.67 | -16.1055 |
|  | XP\_065066977\_5065-model2.68 | -16.1055 |
|  | XP\_065066977\_5065-model2.69 | -16.1055 |
|  | XP\_065066977\_5065-model2.70 | -16.1055 |
|  | XP\_065066977\_5065-model2.71 | -16.0394 |
|  | XP\_065066977\_5065-model2.72 | -16.0394 |
|  | XP\_065066977\_5065-model2.73 | -16.0394 |
|  | XP\_065066977\_5065-model2.74 | -16.0394 |
|  | XP\_065066977\_5065-model2.75 | -16.0394 |
|  | XP\_065066977\_5065-model2.76 | -16.0394 |
|  | XP\_065066977\_5065-model2.77 | -16.0394 |
|  | XP\_065066977\_5065-model2.78 | -16.0394 |
|  | XP\_065066977\_5065-model2.79 | -16.0394 |
|  | XP\_065066977\_5065-model2.80 | -16.0394 |
|  | XP\_065066977\_5065-model2.81 | -16.0377 |
|  | XP\_065066977\_5065-model2.82 | -16.0377 |
|  | XP\_065066977\_5065-model2.83 | -16.0377 |
|  | XP\_065066977\_5065-model2.84 | -15.9121 |
| # cluster 3 - Size: 9 |  |  |
|  | XP\_065066977\_5065-model3 | -16.09 |
|  | XP\_065066977\_5065-model3.1 | -16.09 |
|  | XP\_065066977\_5065-model3.2 | -16.09 |
|  | XP\_065066977\_5065-model3.3 | -16.0542 |
|  | XP\_065066977\_5065-model3.4 | -16.0542 |
|  | XP\_065066977\_5065-model3.5 | -16.0542 |
|  | XP\_065066977\_5065-model3.6 | -16.0542 |
|  | XP\_065066977\_5065-model3.7 | -16.0542 |
|  | XP\_065066977\_5065-model3.8 | -16.0542 |
| # cluster 4 - Size: 2 |  |  |
|  | XP\_065066977\_5065-model4 | -15.9351 |
|  | XP\_065066977\_5065-model4.1 | -15.9351 |
|  |  |  |
